# An Improved Toeplitz Approximation Method for Coherent DOA Estimation in Impulsive Noise Environments

**DOI:** 10.3390/e25060960

**Published:** 2023-06-20

**Authors:** Jiang’an Dai, Tianshuang Qiu, Shengyang Luan, Quan Tian, Jiacheng Zhang

**Affiliations:** 1 Faculty of Electronic Information and Electrical Engineering, Dalian University of Technology, Dalian 116024, China; 2 School of Electrical Engineering and Automation, Jiangsu Normal University, Xuzhou 221116, China; 3 School of Electronics and Information Engineering, Taizhou University, Taizhou 318000, China; tianquan10@163.com; 4 School of Artificial Intelligence, Nanjing University of Information Science and Technology, Nanjing 210000, China

**Keywords:** direction of arrival estimation, coherent signals, impulsive noise, correntropy, generalized covariance, Toeplitz approximation

## Abstract

Direction of arrival (DOA) estimation is an important research topic in array signal processing and widely applied in practical engineering. However, when signal sources are highly correlated or coherent, conventional subspace-based DOA estimation algorithms will perform poorly due to the rank deficiency in the received data covariance matrix. Moreover, conventional DOA estimation algorithms are usually developed under Gaussian-distributed background noise, which will deteriorate significantly in impulsive noise environments. In this paper, a novel method is presented to estimate the DOA of coherent signals in impulsive noise environments. A novel correntropy-based generalized covariance (CEGC) operator is defined and proof of boundedness is given to ensure the effectiveness of the proposed method in impulsive noise environments. Furthermore, an improved Toeplitz approximation method combined CEGC operator is proposed to estimate the DOA of coherent sources. Compared to other existing algorithms, the proposed method can avoid array aperture loss and perform more effectively, even in cases of intense impulsive noise and low snapshot numbers. Finally, comprehensive Monte-Carlo simulations are performed to verify the superiority of the proposed method under various impulsive noise conditions.

## 1. Introduction

As a fundamental component of array signal processing, direction of arrival (DOA) estimation has been continuously attracting much attention from researchers and been widely applied in numerous fields such as radar, sonar, satellite navigation, wireless communications, and biomedicine [1,2,3,4,5]. After decades of research, various high-resolution DOA estimation methods have been proposed [6]. Among them, the subspace-based methods are well-known and effective. Utilizing eigenvalue decomposition (EVD) or singular value decomposition (SVD), the subspace-based methods can partition received data covariance matrices into signal subspace and noise subspace. Based on specific properties of subspace, such as orthogonality and rotational invariance, many high-resolution methods have been proposed, such as multiple signal classification (MUSIC) [7], estimation of signal parameters via rotational invariance techniques (ESPRIT) [8] and subspace fitting methods [9].

Compared to other conventional DOA estimation methods such as the maximum likelihood (ML) methods [10], the subspace-based methods are relatively computationally efficient and highly practical. Thus, the subspace-based methods have been widely studied and many variants have been proposed over the past several years. However, the subspace-based methods were originally proposed for uncorrelated signals for which the rank of the data covariance matrix is equal to the number of signal sources. When the signal sources are highly correlated or coherent, these methods will encounter significant difficulties. Signal coherence is a common phenomenon that can be caused by natural multipath propagation effects or intentional hostile jamming. This will in turn result in a rank deficiency in the source covariance matrix and a divergence of the signal eigenvector into the noise subspace. Therefore, conventional subspace-based methods may produce several pseudo peaks and fail to accurately perform DOA estimation, particularly when the signal sources are close together.

To eliminate the adverse effects caused by signal coherence, numerous methods have been proposed [11,12,13,14,15,16,17,18,19,20,21,22,23,24,25,26,27]. A well-known method known as spatial smoothing (SS) or forward-only spatial smoothing (FOSS) was proposed in Evans et al. [11] to estimate DOA regardless of the coherence of signal sources, and a detailed analysis of the SS method was given in Shan et al. [12]. The main concept of the SS technique is to partition the entire received array into several overlapped subarrays and obtain a modified signal covariance matrix through subarray averaging. The SS technique can be seen as a preprocessing procedure to de-correlate the received signals and resolve DOA estimation of coherent sources when combined with the subspace-based methods. However, the SS technique does not fully utilize the useful information in the signal covariance matrix. Moreover, the effective aperture of the array is significantly reduced due to the subarray’s partition and average operation, which means the number of coherent signals that can be detected is significantly reduced. To circumvent these problems, several modified SS methods have been proposed [13,14,15,16,17,18,19]. In Williams et al. [13], an improved SS method called forward/backward spatial smoothing (FBSS) was developed to reduce aperture loss. The FBSS method utilizes both forward subarrays and complex conjugated backward subarrays to improve the performance and is able to estimate any *K* DOAs using 3*K*/2 sensor elements, while the SS method requires 2*K* sensor elements by contrast. Li [15] examined the performance of the SS as well as the FBSS method and showed that the angular resolution can be enhanced by squaring array covariance matrices. In Du and Kirlin [17], an improved spatial smoothing method that can fully utilize the correlations of array outputs was presented. Pan et al. [19] proposed an enhanced spatial smoothing (ESS) technique that fully exploits the information of both the covariance matrices and cross-covariance matrices of the subarrays. Although these modified SS methods can improve the performance of the SS method to a certain extent, the problem of aperture loss remains unresolved.

In addition to the SS-based methods, the Toeplitz approximation method (TAM) [20] is an alternative to circumvent problems encountered in the DOA estimation of coherent signals. The TAM method is proposed based on the fact that the covariance matrix of uncorrelated stationary sources is Toeplitz and can reconstruct the covariance matrix of coherent sources with Toeplitz structure. Compared to the SS method, the TAM method does not reduce the effective aperture of the array and has higher resolution capability. In Chen et al. [22], a modification of the TAM and an iterative version of the TAM were used for bearing estimation with sensor location errors. Han et al. [23] proposed an ESPRIT-like algorithm based on Toeplitz matrix reconstruction. This method can achieve accurate estimation performance with low computational complexity; however, the method requires additional array elements. In Qian et al. [24], a coherent DOA estimation scheme was proposed to solve the optimization problem based on a newly designed cost function. This scheme can adequately exploit the information of the reconstructed Toeplitz matrix and work properly without a priori information, such as the source number. Zhang et al. [25] proposed a multiple Toeplitz matrices reconstruction method for coherent DOA estimation that adequately applied information contained in the correlation matrices. In Zhang et al. [26], a modified method called forward and backward partial Toeplitz matrices reconstruction (FB-PTMR) was proposed, which exploits half of the array covariance matrix to reconstruct data in a Toeplitz matrix. The FB-PTMR can overcome the drawbacks of other methods due to its neglect of phase differences and utilize more information compared to the ESPRIT-like algorithm. Dai et al. [27] proposed a coherent DOA estimation scheme combining full-row Toeplitz matrices reconstruction and deep learning network architecture to achieve higher performance with lower computational cost.

The coherent DOA estimation methods mentioned above assume that ambient noise is Gaussian-distributed. In reality, however, there are various non-Gaussian noises with spike impulse characteristics [28,29,30], such as atmospheric noise, underwater noise, vehicle ignition, multi-user interference, etc. These impulsive noises usually have heavy-tailed distributions, which means the probability density function (PDF) decreases more slowly and outliers are more likely to occur compared to Gaussian distribution. Under the impulsive noise environment, the performance of the above coherent DOA estimation methods will severely degrade. Therefore, an appropriate distribution method is needed to model the impulse noise. There are multiple non-Gaussian distributions used to characterize impulsive noise with heavy-tailed PDFs, such as generalized Gaussian distribution [31], Gaussian mixture distribution [32], and alpha-stable (*α*-stable) distribution [28,33]. Among these distributions, only *α*-stable distribution satisfies the generalized central limit theorem [33]. Meanwhile, *α*-stable distribution could provide a connection between Gaussian and non-Gaussian distribution. In fact, Gaussian distributions and various non-Gaussian distributions are special cases of the *α*-stable distribution family. Therefore, *α*-stable distribution is an appropriate model for impulsive noise.

Some studies have been devoted to the problem of coherent DOA estimation in the presence of impulsive noise. In Visuri et al. [34], two nonparametric DOA estimation algorithms were presented in the presence of non-Gaussian noise and multipath propagation effects. These algorithms are based on multivariate spatial sign covariance matrices and combined with SS to deal with coherent sources. The theoretical analysis and some extensions of the algorithms are then given in Visuri et al. [35]. Rupi et al. [36] combined a signed-power nonlinearity and SS to mitigate the effects of the heavy-tailed background noise and reduce the measured coherence. In Li et al. [37], novel algorithms based on fractional lower-order statistics (FLOS) and FBSS were proposed for DOA estimation of coherent sources in the presence of impulsive noise. Liu et al. [38] presented an algorithm which combined SS and infinity-norm normalization (INF) to estimate the DOA of coherent sources in impulsive noise environments. In Li and Lin [39], SS was applied to the phased fractional lower order moments (PFLOM) matrices and a robust algorithm called PFLOM-SS was proposed for DOA estimation of coherent sources under impulsive noise environments. Guan et al. [40] defined and compared three different decorrelation methods for DOA estimation of coherent sources based on correntropy-based correlation (CRCO). These improved SS-based methods can alleviate performance degradation to a certain extent, but they share the inherent disadvantages of the SS-based algorithm, that is, the effective aperture array will be reduced. Meanwhile, the performance of these methods will deteriorate significantly when the ambient noise is highly impulsive, and the number of snapshots is low. Therefore, further studies are required to adequately address the problem of DOA estimation of coherent signals under impulsive noise.

In this paper, a new coherent DOA estimation method based on correntropy-based generalized covariance (CEGC) and Toeplitz approximation are presented. The remainder of this paper is organized as follows. Section 2 presents some preliminary knowledge related to our method, including the signal model and noise model. In Section 3, the procedure of the proposed method is briefly introduced. In Section 4, Monte-Carlo simulations are carried out to evaluate the performance of the proposed and other existing methods under different conditions. Conclusions are finally drawn in Section 5.

Notation: Matrices, vectors, and scalar quantities are denoted by uppercase boldface, lowercase boldface, and lowercase letters, respectively. ${\left(\cdot \right)}^{*}$, ${\left(\cdot \right)}^{T}$, and ${\left(\cdot \right)}^{H}$ denote conjugate, transpose, and conjugate transpose, respectively. diag(·) denotes diagonal matrix. δ(·) denotes the Dirac delta function. |·| denotes absolute value operation. max(·) denotes maximum operation. E(·) denotes the mathematic expectation operation.

## 2. Preliminaries

### 2.1. Signal Model of Coherent DOA Estimation

Consider a uniform linear array (ULA) consisting of *M* omnidirectional sensors receiving *K* narrow-band plane-wave signals from directions $\left\{\theta_{1},\theta_{2},\ldots ,\theta_{K}\right\}$, where $\theta_{k}$ denotes the DOA of the *k*th signal source.

Due to the effect of multipath propagation and other factors, there is usually a certain degree of correlation between two signal sources. The correlation coefficient $\rho_{ij}$ between $x_{i}(t)$ and $x_{j}(t)$ indicates the degree of correlation and can be written as:

(1) $$\rho_{ij}=\frac{E\left[x_{i}(t)x_{j}^{*}(t)\right]}{\sqrt{E\left[{\left|x_{i}(t)\right|}^{2}\right]E\left[{\left|x_{j}(t)\right|}^{2}\right]}}$$

According to the Cauchy–Schwarz inequality, we know that $\left|\rho_{ij}\right|\leq 1$. When $\rho_{ij}=0$, $x_{i}(t)$ and $x_{j}(t)$ are uncorrelated. When $0<\left|\rho_{ij}\right|<1$, $x_{i}(t)$ and $x_{j}(t)$ are (partially) correlated. When $\left|\rho_{ij}\right|=1$, $x_{i}(t)$ and $x_{j}(t)$ are coherent (completely correlated).

If the *K* signal sources $\left\{x_{1}(t),x_{2}(t),\ldots ,x_{K}(t)\right\}$ are coherent, taking the first signal $x_{1}(t)$ as the reference, the *k*th signal source at time *t* can be represented as

(2) $$x_{k}(t)=A_{k}x_{1}(t),k=1,2,\ldots ,K$$

where $A_{k}$ denotes the complex attenuation of the *k*th signal with respect to $x_{1}(t)$. Then the signal sources matrix can be represented as:

(3) $$X=\left[x(1),x(2),\ldots ,x(N)\right]$$

where *N* is the number of snapshots. $x(t)={\left[x_{1}(t),x_{2}(t),\ldots ,x_{K}(t)\right]}^{T}$ represents the signal sources vector.

The array manifold matrix can be represented as:

(4) $$A=\left[a\left(\theta_{1}\right),a\left(\theta_{2}\right),\ldots ,a\left(\theta_{K}\right)\right]$$

where $a\left(\theta_{k}\right)={\left[a_{1}\left(\theta_{k}\right),a_{2}\left(\theta_{k}\right),\ldots ,a_{M}\left(\theta_{k}\right)\right]}^{T}$ represents the steering vector of the *k*th signal source. $a_{m}\left(\theta_{k}\right){=e}^{j2\pi \sin\theta_{k}\left(m-1\right)d/\lambda }$ represents the component of $a\left(\theta_{k}\right)$ corresponding to the *m*th sensor. d is the spacing between two adjacent sensors. λ is the wavelength.

Using complex signal representation, the received signal $y_{m}(t)$ at the *m*th sensor can be represented as:

(5) $$y_{m}(t)=\sum_{k=1}^{K}x_{k}(t)a_{m}\left(\theta_{k}\right)+w_{m}(t),\;m=1,2,\ldots ,M$$

where $w_{m}(t)$ represents the noise at the *m*th sensor.

Then the received signal matrix can be represented as:

(6) $$Y=\left[y(1),y(2),\ldots ,y(N)\right]$$

where $y(t)={\left[y_{1}(t),y_{2}(t),\ldots ,y_{M}(t)\right]}^{T}$ represents the received signal vector.

Moreover, Equation (5) can be represented more compactly in matrix form as:

(7) Y=AX+W

where $W=\left[w(1),w(2),\ldots ,w(N)\right]$ denotes the received noise matrix. $w(t)={\left[w_{1}(t),w_{2}(t),\ldots ,w_{M}(t)\right]}^{T}$ represents the received noise vector. The purpose of this paper is utilizing noisy received data Y to estimate the DOA $\left\{\theta_{1},\theta_{2},\ldots ,\theta_{K}\right\}$ of *K* coherent signal sources $\left\{x_{1}(t),x_{2}(t),\ldots ,x_{K}(t)\right\}$.

### 2.2. α-Stable Distribution Noise Model

The *α*-stable distribution is an appropriate model of non-Gaussian impulsive noise due to its generality. Since there is no closed-form expression for its PDF, *α*-stable distribution is usually described by its characteristic function. The characteristic function of α-stable distribution can be represented as in Shao and Nikias [33]:

(8) $$\phi \left(\omega \right)=e^{j\mu \omega -\gamma {\left|\omega \right|}^{\alpha }\left[1+j\beta \mathrm{sgn}\left(\omega \right)\zeta \left(\omega ,\alpha \right)\right]}$$

(9) $$\mathrm{sgn}\left(\omega \right)=\left\{\begin{array}{c} 1\\ 0\\ -1 \end{array}\right.\begin{array}{c} ,\\ ,\\ , \end{array}\begin{array}{c} \omega >0\\ \omega =0\\ \omega <0 \end{array}$$

(10) $$\zeta \left(\omega ,\alpha \right)=\left\{\begin{array}{c} \tan\frac{\pi \alpha }{2},\alpha \neq 1\\ \frac{2}{\pi }\log\left|\omega \right|,\alpha =1 \end{array}\right.$$

where μ∈(−∞,+∞) is the location parameter. α∈(0,2] is the characteristic exponent, and it measures the thickness of the tails of the PDF. When α=2, *α*-stable distribution is equivalent to Gaussian distribution. With the decrease of α, the noise will behave more impulsively. β∈[−1,1] is the symmetry parameter. When β=0, the distribution is symmetric about the center μ and is called symmetric alpha-stable (*SαS*) distribution. γ∈(0,+∞) is the dispersion parameter and plays a role similar to that of the variance for Gaussian distribution.

In this paper, we utilize the *SαS* distribution to model impulsive noise. Therefore, (8) can be simplified as:

(11) $$\phi \left(\omega \right)=e^{j\mu \omega -\gamma {\left|\omega \right|}^{\alpha }}$$

## 3. Methodology

In this paper, we focus on estimating the DOAs $\left\{\theta_{1},\theta_{2},\ldots ,\theta_{K}\right\}$ of coherent signals from the received array data matrix, Y, contaminated by *α*-stable distribution noise. First, a novel operator called CEGC is defined. The boundedness of the CEGC operator is proven to ensure the effectiveness of the proposed method. Subsequently, a coherent DOA estimation method based on CEGC and Toeplitz approximation is derived in detail. The major implementation steps of our proposed method are listed at the end of this section.

### 3.1. CEGC

Conventional subspace-based DOA estimation methods are based on EVD or SVD of the data covariance matrix, which will fail under *α*-stable distribution noise since the data statistics of order greater than or equal to two are unbounded in this environment. To overcome this drawback, several modified operators are proposed, of which the FLOS [33] is a typical example. However, the FLOS-based methods rely on prior knowledge of the impulsive noise to select appropriate parameter and require large sample sizes to meet a satisfactory performance. This motivates us to develop more effective operators to deal with impulsive noise.

Recently, a local similarity measurement called correntropy has been proposed [41,42] and has become popular in the area of non-Gaussian signal processing [43,44,45]. Compared to the FLOS, correntropy can exploit more intrinsic information about infinite statistical moments of the signal and deal with outliers without prior knowledge of the noise. The correntropy of two random variables *X* and *Y* is defined as:

(12) $$C_{\sigma }\left(X,Y\right)=E\left[\kappa_{\sigma }\left(X-Y\right)\right]$$

where $\kappa_{\sigma }\left(\cdot \right)$ represents the kernel function and σ is the kernel size. Utilizing data samples ${\left\{\left(x_{i},y_{i}\right)\right\}}_{i=1}^{N}$, correntropy can be estimated as follows:

(13) $${\hat{C}}_{\sigma }\left(X,Y\right)=\frac{1}{N}\sum_{i=1}^{N}\kappa_{\sigma }\left(x_{i}-y_{i}\right)$$

As a similarity measure between two random variables, correntropy can be regarded as a generalization of the conventional Pearson correlation [41]. Introducing nonlinear mapping by kernel function, correntropy can transform data from the input space to an infinite dimensional, reproducing kernel Hilbert space and effectively eliminating the adverse effect of outliers.

Moreover, the concept of generalized covariance (GC) [46] is introduced to improve the DOA estimation performance in the presence of α-stable distribution noise, since conventional covariance does not converge in this environment. In fact, a series of existing concepts such as FLOS, hyperbolic tangent, and correntropy can unify in the name of GC. The GC of two random variables, *X* and *Y,* is defined as:

(14) $$R^{\mathrm{GC}}\left(X,Y\right)=E\left[\frac{g_{1}\left(X\right)}{h_{1}\left(X,Y\right)}\cdot \frac{g_{2}\left(X\right)}{h_{2}\left(X,Y\right)}\right]$$

where $g_{1}\left(\cdot \right)$ and $g_{2}\left(\cdot \right)$ represent single-variable functions. $h_{1}\left(\cdot ,\cdot \right)$ and $h_{2}\left(\cdot ,\cdot \right)$ represent dual-variable functions, however, both can also be single-variable functions or even constants in some cases.

Inspired by the advantages of correntropy and GC, a novel operator called CEGC is proposed and defined as follows:

(15) $$\bar{R}=E\left[e^{-\frac{\left|X\right|+\left|Y\right|}{\sigma }}XY\right]$$

where the kernel size σ>0.

The CEGC operator retains the conventional correlation term and utilizes the exponential kernel to provide further outlier suppression capabilities to adapt to an intensive impulsive noise environment. Furthermore, it can be regarded as a specific case of GC based on the generalized exponential kernel function. In this case, $g_{1}\left(X\right)=X$, $g_{2}\left(Y\right)=Y$, $h_{1}\left(\cdot ,\cdot \right)=e^{-\left|X\right|/\sigma }$, $h_{2}\left(\cdot ,\cdot \right)=e^{-\left|Y\right|/\sigma }$. To ensure the effectiveness of the proposed method, we prove the boundedness of the CEGC operator.

**Theorem** **1.** *If X and Y are two independent and identically distributed SαS random variables, the CEGC between X and Y is bounded*.

**Proof** **of** **Theorem** **1.** We can obtain the CEGC between *X* and *Y* as Equation (15). It is apparent that $XY\leq {\left[\max\left(\left|X\right|,\left|Y\right|\right)\right]}^{2}$. Assuming $\left|X\right|\geq \left|Y\right|$, we obtain:

(16) $$\begin{array}{ll} \left|\bar{R}\right| & \leq E\left[e^{-\frac{\left|X\right|+\left|Y\right|}{\sigma }}{\left|X\right|}^{2}\right]\\ & \leq E\left[e^{-\frac{\left|X\right|}{\sigma }}{\left|X\right|}^{2}\right] \end{array}$$

Substituting the characteristic function (11) into (16), we obtain:

(17) $$\begin{array}{ll} \left|\bar{R}\right| & \leq E\left[e^{-\frac{\left|X\right|}{\sigma }}{\left|X\right|}^{2}\right]\\ & =\frac{1}{2\pi }\int_{-\infty }^{+\infty }\int_{-\infty }^{+\infty }e^{-\frac{\left|X\right|}{\sigma }}{\left|X\right|}^{2}e^{j\mu \omega -\gamma {\left|\omega \right|}^{\alpha }}e^{-j\omega X}dXd\omega \\ & \leq \frac{1}{\pi }\int_{0}^{+\infty }\int_{-\infty }^{+\infty }e^{-\frac{X}{\sigma }}X^{2}e^{-\gamma {\left|\omega \right|}^{\alpha }}d\omega dX\\ & =\frac{1}{\pi }\int_{0}^{+\infty }e^{-\frac{X}{\sigma }}X^{2}dX\int_{-\infty }^{+\infty }e^{-\gamma {\left|\omega \right|}^{\alpha }}d\omega \end{array}$$

where $\int_{-\infty }^{+\infty }e^{-\gamma {\left|\omega \right|}^{\alpha }}d\omega =2\int_{0}^{+\infty }e^{-\gamma \omega^{\alpha }}d\omega =\frac{2}{\alpha }\gamma^{-\frac{1}{\alpha }}\Gamma \left(\frac{1}{\alpha }\right)=h$ and $\Gamma \left(x\right)=\int_{0}^{+\infty }t^{x-1}e^{-t}dt$ denotes the gamma function.

Next, (17) can be simplified as:

(18) $$\begin{array}{ll} \left|\bar{R}\right| & \leq \frac{h}{\pi }\int_{0}^{+\infty }e^{-\frac{X}{\sigma }}X^{2}dX\\ & =\frac{2h\sigma^{3}}{\pi }<+\infty \end{array}$$

According to (18), we can infer that the CEGC between *X* and *Y* is bounded. Thus ends the proof. □

In the next section, the CEGC is utilized to estimate the DOA rather than conventional correlation in the covariance matrix. 

### 3.2. Proposed Method

According to the received signal model (7) and assuming that the noise is independent of the signals, the data covariance matrix, $R_{Y}$, can be written as:

(19) $$\begin{array}{ll} R_{Y} & =E\left\{YY^{H}\right\}\\ & =AE\left\{XX^{H}\right\}A^{H}+E\left\{WW^{H}\right\}\\ & =AR_{X}A^{H}+E\left\{WW^{H}\right\} \end{array}$$

Conventional subspace-based methods must perform EVD or SVD of the data covariance matrix. When the signal sources are coherent, the source covariance matrix, $R_{X}$, is rank deficient, i.e., $R_{X}$ is a singular matrix and the rank of $R_{X}$ is lower than the number of signal source, *K*. Therefore, conventional subspace-based methods cannot produce accurate DOA estimations and modified solutions should be used to resolve this situation.

Compared with SS-based methods, the TAM [20,21,22,23,24,25,26,27] method is an effective alternative to resolve coherent signals, since it does not reduce the effective aperture of the array and is capable of achieving higher resolution. However, these methods will fail under *α*-stable distributed noise because they are developed based on second-order statistics, which are unbounded under *α*-stable distributed noise.

Combined with the operator CEGC described in Section 3.1, we can construct the pseudo-covariance matrix, ${\bar{R}}_{Y}$, of the received signal, Y, as:

(20) $${\bar{R}}_{Y}=\left[\begin{array}{cccc} {\bar{R}}_{1,1} & {\bar{R}}_{1,2} & \cdots & {\bar{R}}_{1,M}\\ {\bar{R}}_{2,1} & {\bar{R}}_{2,2} & \cdots & {\bar{R}}_{2,M}\\ \vdots & \vdots & \ddots & \vdots \\ {\bar{R}}_{M,1} & {\bar{R}}_{M,2} & \cdots & {\bar{R}}_{M,M} \end{array}\right]$$

where:

(21) $${\bar{R}}_{i,j}=\bar{R}\left(y_{i},y_{j}{}^{*}\right)=\frac{1}{N}\sum_{n=1}^{N}e^{-\frac{\left|y_{i}\right|+\left|y_{j}{}^{*}\right|}{\sigma }}y_{i}y_{j}{}^{*}$$

Furthermore, to circumvent problems due to signal coherence, we perform Toeplitz approximation on ${\bar{R}}_{Y}$ and construct a Toeplitz matrix, $R_{\mathrm{Top}}$, as:

(22) $$R_{\mathrm{Top}}=\left[\begin{array}{cccc} R_{\mathrm{Top}}\left(0\right) & R_{\mathrm{Top}}\left(1\right) & \cdots & R_{\mathrm{Top}}\left(M-1\right)\\ R_{\mathrm{Top}}\left(-1\right) & R_{\mathrm{Top}}\left(0\right) & \cdots & R_{\mathrm{Top}}\left(M-2\right)\\ \vdots & \vdots & \ddots & \vdots \\ R_{\mathrm{Top}}\left(1-M\right) & R_{\mathrm{Top}}\left(2-M\right) & \cdots & R_{\mathrm{Top}}\left(0\right) \end{array}\right]$$

where:

(23) $$R_{\mathrm{Top}}\left(m\right)=\left\{\begin{array}{l} \frac{1}{M-m}\sum_{i=1}^{M-m}{\bar{R}}_{i,i+m},\;0\leq m<M\\ \frac{1}{M+m}\sum_{i=1}^{M+m}{\bar{R}}_{i-m,i},\;-M<m\leq 0 \end{array}\right.$$

Inspired by the SS-based methods, the FBSS technique can be applied for effective utilization of the data matrix, and we obtain:

(24) $${\bar{R}}_{\mathrm{Top}}=\frac{1}{2}\left(R_{\mathrm{Top}}+J_{M}R_{\mathrm{Top}}^{*}J_{M}\right)$$

where $J_{M}$ denotes the exchange matrix whose components are zeros except for components on the anti-diagonal. Meanwhile, iterative algorithms [21,22] can also be applied to estimate ${\bar{R}}_{\mathrm{Top}}$ to further improve the performance of the proposed method.

By performing EVD of ${\bar{R}}_{\mathrm{Top}}$, we obtain:

(25) $${\bar{R}}_{\mathrm{Top}}=U_{S}\Sigma_{S}U_{S}^{H}+U_{N}\Sigma_{N}U_{N}^{H}$$

where $\Sigma_{S}$ and $\Sigma_{N}$ are the diagonal matrices with the *K* largest eigenvalues and the remaining M−K smaller eigenvalues of ${\bar{R}}_{\mathrm{Top}}$, respectively. $U_{S}$ and $U_{N}$ are the matrices composed of eigenvectors corresponding to $\Sigma_{S}$ and $\Sigma_{N}$, and span the signal and noise subspace of ${\bar{R}}_{\mathrm{Top}}$, respectively.

Furthermore, conventional subspace-based methods can be applied to estimate the DOA. We can construct the spatial spectrum based on the classical MUSIC algorithm as:

(26) $$S_{\mathrm{Top}}\left(\theta \right)=\frac{1}{a^{H}\left(\theta \right)U_{N}U_{N}^{H}a\left(\theta \right)},\;-\frac{\pi }{2}\leq \theta \leq \frac{\pi }{2}$$

By searching *K* largest peaks of (26), we obtain the DOA estimation $\left\{{\hat{\theta }}_{1},{\hat{\theta }}_{2},\ldots ,{\hat{\theta }}_{K}\right\}$. At this point, we have achieved the proposal for coherent DOA estimation under impulsive noise. The major implementation steps of our proposed method are summarized as follows:

**Step** **1:** Use the array received signal matrix (6) as input to construct the pseudo-covariance matrix, ${\bar{R}}_{Y}$ based on (20) and (21).
**Step** **2:** Perform Toeplitz approximation on ${\bar{R}}_{Y}$ based on (22) and (23) to construct a Toeplitz matrix, $R_{\mathrm{Top}}$.
**Step** **3:** Construct a modified matrix, ${\bar{R}}_{\mathrm{Top}}$ based on (24).
**Step** **4:** Perform the EVD of ${\bar{R}}_{\mathrm{Top}}$ to obtain the eigenvectors, $U_{N}$, corresponding to the noise subspace.
**Step** **5:** Calculate the spatial spectrum function (26) and search *K* largest peaks to estimate the DOA of coherent sources.

## 4. Simulation

In this section, the DOA estimation performance of the proposed method will be evaluated through comprehensive simulations. The simulation results will be analyzed in detail.

We consider a ULA with M=10 sensors whose inter-element spacings are set as a half wavelength. Assume *K* coherent narrow band signals are located in the far-field of arrays. The number of snapshots is N=500 except in Section 4.4.

Since the typical signal-to-noise ratio will diverge under *SαS* noise, the generalized signal-to-noise ratio (GSNR) [28] is employed to measure noise intensity and is defined as:

(27) $$\mathrm{GSNR}=10\log_{10}\frac{P_{s}}{\gamma }$$

where $P_{s}$ is the power of signal and *γ* is the dispersion parameter of *SαS* noise.

The proposed method is compared to the FBSS method [13], TAM method [20], FLOM-SS method [37], PFLOM-SS method [39], and CRCO-SS method [40]. For SS-based methods, the number of sensors in the subarray is $M_{1}=6$. The performance of different algorithms is evaluated by two quantities called probability of resolution (PR) and root-mean-square error (RMSE).

In the experiments, a successful resolution of DOA is defined as:

(28) $$\left|{\hat{\theta }}_{k}\left(l\right)-\theta_{k}\right|\leq 2^{{}^{\circ}},k=1,\ldots ,K$$

where ${\hat{\theta }}_{k}\left(l\right)$ are the estimated DOA values of the *k*th target in the *l*th Monte-Carlo trial. Therefore, the PR is defined as the ratio of the number of successful resolutions to the number of Monte-Carlo trials.

The RMSE is defined as follows:

(29) $$\mathrm{RMSE}=\sqrt{\frac{1}{KL}\sum_{k=1}^{K}\sum_{l=1}^{L}{\left|{\hat{\theta }}_{k}\left(l\right)-\theta_{k}\right|}^{2}}$$

where *L* is the number of Monte Carlo trials. Unless otherwise stated, every simulation will carry out 200 Monte Carlo trials.

The related parameters for the simulations are listed in Table 1.

### 4.1. Spatial Spectrums Comparison

**Figure 1:**
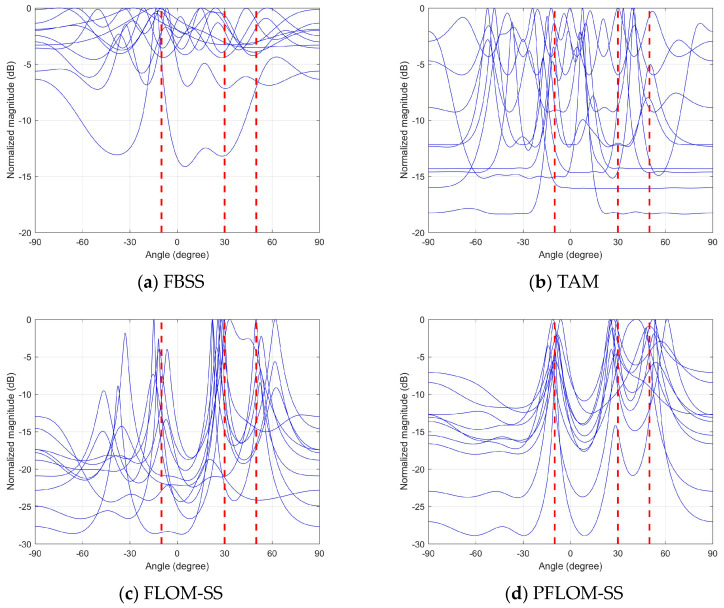

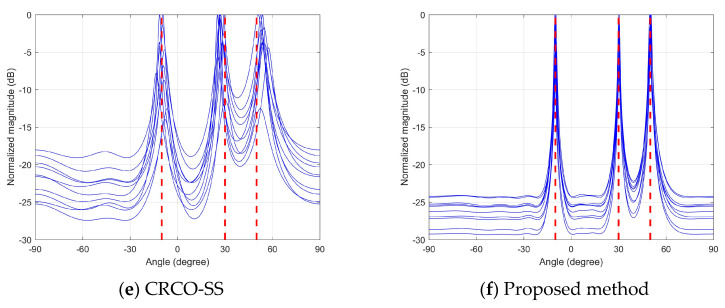
Spatial spectrograms comparison.

In this simulation, we will compare the spectrograms of six candidate methods under a relatively severe *SαS* noise environment (α=1.3, GSNR=0 dB). Three coherent signals are located at $\left(-10^{{}^{\circ}},30^{{}^{\circ}},50^{{}^{\circ}}\right)$, which are represented by red dashed lines in Figure 1. The spectrograms of FBSS, TAM, FLOM-SS, PFLOM-SS, CRCO-SS, and the proposed method are shown in Figure 1a–f, respectively. Every figure contains 10 Monte Carlo trials of spatial spectrum.

Figure 1a,b are the spectrograms of FBSS and TAM, respectively. As expected, these two algorithms cannot function properly in the presence of impulsive noise. Three DOAs can hardly be resolved. The FLOM-SS, PFLOM-SS, and CRCO-SS methods are shown in Figure 1c–e, respectively. They can alleviate the impact of impulse noise on DOA estimation. However, the accuracy and stability of the algorithms must be improved. Meanwhile, it can be seen from Figure 1f that the proposed method has the highest performance spectrograms and all DOAs can be resolved easily.

In the following simulations, more numerical results will be given to compare these methods.

### 4.2. Experiment Results vs. GSNRs

**Figure 2:**
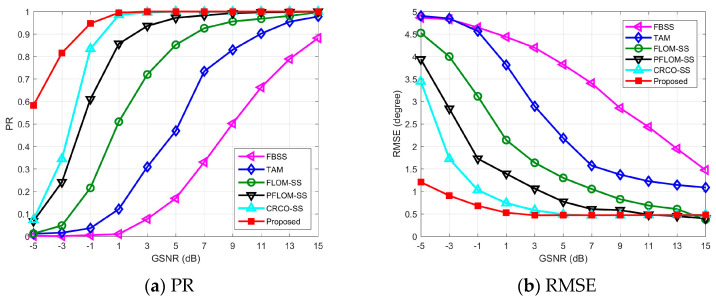
Experimental results vs. GSNRs.

This simulation will focus on the performance comparison with a wide range of GSNRs. Experiment results can be found in Figure 2. Detailed parameters can be found in Table 1.

The PR of all candidate methods are shown in Figure 2a. We find that the performances of PR are improved with the increase of GSNRs. For moderate impulsive noise environments (GSNR>5 dB), most methods show effective performances except for SS and TAM methods. These two methods are developed based on Gaussian noise assumptions and cannot accurately estimate DOA under impulsive noise. With the decrease of GSNR, the PR of FLOM-SS, PFLOM-SS, and CRCO-SS decrease rapidly. When GSNR=−5 dB, the PR of these three methods are all below 0.1. This indicates that the SS-variant methods cannot resolve DOA in intense impulsive noise environment. Meanwhile, the proposed method has relatively high PR, even when GSNR is fairly low.

To further explore the performance, the RMSE of DOA estimation using different methods are compared in Figure 2b. The FBSS and TAM methods can achieve low RMSE results only when GSNR is high enough. The RMSE of three SS-variant methods increase rapidly when GSNR<0 dB. Among all candidate methods, the proposed method has the most effective RMSE result.

### 4.3. Experiment Results vs. Characteristic Exponents α

**Figure 3:**
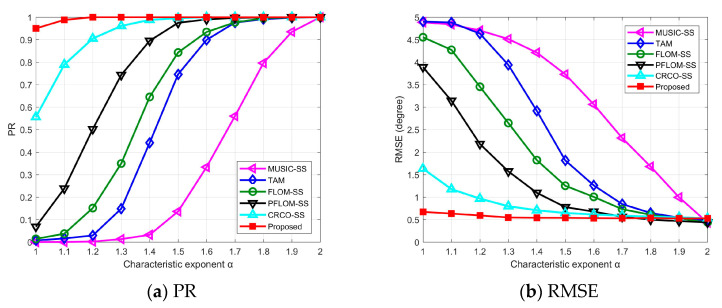
Experimental results vs. characteristic exponent *α*.

This simulation will focus on the performance comparison with a wide range of the characteristic exponent *α*. PR and RMSE results can be found in Figure 3. Detailed parameters can be found in Table 1.

From Figure 3a, we can conclude that all candidate methods can successfully resolve the DOA when α=2, which indicates the validity of all methods under Gaussian noise. With the decrease of *α*, the impulsiveness of *SαS* noise is gradually enhanced and the performance of FBSS and TAM deteriorate significantly. The FLOM-SS and PFLOM-SS can achieve high PR when α>1.5 and degrade severely when *α* approaches 1.0. This is because FLOS-based methods need prior knowledge of the impulsive noise for a satisfactory performance and cannot resist intense impulsive noise. The proposed method outperforms other candidate methods and has the most effective PR result.

Figure 3b is the RMSE results of candidate methods. We find that the RMSE curve of the proposed method fluctuates slightly even when *α* approaches 1.0. Three SS-variant methods can work well in moderately impulsive noise environments. The RMSE of FBSS and TAM methods increase rapidly with the decrease of *α*, which shows their vulnerability in impulsive noise environments.

### 4.4. Experiment Results vs. Number of Snapshots

**Figure 4:**
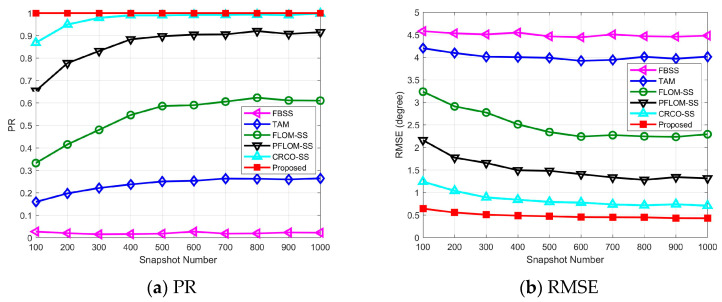
Experimental results vs. number of snapshots.

In this simulation, we evaluate the algorithm performance against the number of snapshots, where α=1.3 and GSNR=0 dB are set. Experiment results can be found in Figure 4. Detailed parameters can be found in Table 1.

As expected, the FBSS and TAM algorithm cannot function properly in the presence of impulsive noise and the number of snapshots has little effect on their performance. For FLOM-SS, PFLOM-SS, and CRCO-SS, the performance improves with the increase of snapshots and towards stability when the number of snapshots is greater than 500. This indicates that a certain number of snapshots are needed to reach satisfactory performance of the algorithms. Meanwhile, the proposed method requires fewer snapshots to achieve stable performance and the results of PR and RMSE outperform other candidate methods.

## 5. Conclusions

In this paper, we have considered the problem of coherent DOA estimation under *α*-stable distributed noise. A novel operator termed CEGC was defined first. To ensure the effectiveness of the proposed method under *α*-stable distributed noise, the proof of boundedness was also provided. Later, we constructed a modified covariance matrix based on CEGC operators and Toeplitz approximation. The modified covariance matrix can be applied to subspace-based methods to estimate the DOA of coherent sources. Finally, multiple simulations were carried out to evaluate the performance of the different methods. Simulation results demonstrate that the proposed method outperforms other existing methods in various *α*-stable distributed noise environments, even in the case of intense impulsive noise and low snapshot numbers. More array models and computationally efficient methods will be considered in our future works.

## Figures and Tables

**Table 1:** Simulation Conditions and Parameter Settings.

Simulations	DOA (Degree)	GSNR (dB)	*α*	Number of Snapshots
4.1	$\left(-10,30,50\right)$	0	1.3	500
4.2	$\left(10,30\right)$	$\left[-5,15\right]$	1.3	500
4.3	$\left(10,30\right)$	0	$\left[1.0,2.0\right]$	500
4.4	$\left(10,30\right)$	0	1.3	$\left[100,1000\right]$

## Data Availability

The data are available from the corresponding author upon reasonable request.
